# Measurements for the institutional cohesion dimension of the standard model of military group cohesion

**DOI:** 10.1080/08995605.2021.1897491

**Published:** 2021-03-29

**Authors:** Ralph Sundberg, Chiara Ruffa

**Affiliations:** a Department of Peace and Conflict Research, Uppsala University, Uppsala, Sweden; b National Defence University, Stockholm, Sweden

**Keywords:** Cohesion, performance, structural equation models

## Abstract

Notwithstanding the prominence of the so-called Standard Model of Military Group Cohesion (SMMGC), important parts of the model are understudied: both conceptually and empirically. In this article we, first, synthesize previous research to conceptualize and measure the overlooked institutional cohesion dimension. Second, we test the validity of the proposed full four-dimensional SMMGC model using a survey of an Italian Alpini battalion, and more rigorous methods than in previous research. Results are supportive of our proposed measurements and the validity of the four-dimensional model. We thus make a methodological and an empirical contribution to further the ongoing debate on military cohesion.

**What is the public significance of this article?**—Cohesion in military forces is believed to be an important factor for combat groups to stick together during stress. This article studies how we can theorize and measure a so far understudied dimensions of cohesion: bonding with military institutions. Studying approximately 480 members of an Italian infantry unit the study finds that institutional cohesion can be measured in a valid way, yielding support for how to conceptualize and model the broader concept of cohesion.

Cohesion in military forces is often measured using the Standard Model of Military Group Cohesion (SMMGC; Bartone, Johnsen, Eid, Brun, & Laberg, 2002; Salo, 2011; Siebold, 2007, 2011). It has lately been argued – prominently in a special issue on military group cohesion edited by Käihkö (2018) – that despite the proposed importance of cohesion for military outcomes the concept is understudied. Käihkö (2018) identified that dominating psychometric takes have failed to take heed to two important levels of analysis: cohesion on the meso-level of the military institution and the macro-level of state and society. Käihkö (2018) argued that these levels of analysis need to be included to understand cohesion: something argued also by scholars more psychometrically inclined (Salo, 2011; Salo & Siebold, 2008). Within the SMMGC the meso and macro levels are both vaguely conceptualized and rarely measured.

We tackle this oversight by conceptualizing and measuring an important meso-level dimension: institutional cohesion. The contribution of this study thus speaks to concerns raised by Käihkö and others regarding the SMMGC while also expanding upon and further validating the usefulness of Siebold’s cohesion model. We, first, synthesize previous conceptions of institutional cohesion – the SMMGC label that corresponds to the meso-level- to delineate a coherent concept before creating a new measurement battery. Second, we subject cohesion variables – for all four dimensions- to validity and reliability tests using SEM and CFA. These techniques allows us to evaluate the validity and reliability of the full four-dimensional structure of the concept instead of – as in most earlier research – evaluate each dimension separately. Our main contributions are methodological and empirical: the construction of a sound measurement of institutional cohesion and expansion of the applicability of the SMMGC. We also make a conceptual contribution through our synthesis of conceptualizations of the meso/institutional level of cohesion. We conduct our tests via a case study of the 2nd Italian *Alpini* battalion, surveying some 470 service members.

## Theoretical framework: The SMMGC and the pieces we are missing

Building mainly on groundwork laid by Siebold (Siebold, 1987; Siebold & Kelly, 1988) scholars have added to, expanded, and modified the SMMGC and demonstrated good validity, at least within Western militaries (Griffith, 2007; Griffith & Vaitkus, 1999; Salo, 2006, 2011; Salo & Siebold, 2005; Siebold & Lindsay, 1999). The SMMGC defines cohesion as “ … the social relationship, both affective and instrumental, of changeable strength (weak to strong) between service members and their group, organization, and service institution” (Siebold, 2007, p. 288). Affective implies bonds based more on emotions, and instrumental bonds based on exchanges of services. Within this broad definition several separate dimensions and components are theorized. The most important distinction is between primary group and secondary group cohesion. Primary group cohesion is the bond a soldier has with individuals with which he/she has face-to-face relationships, such as the platoon and its direct leaders. Secondary group cohesion refers to individual-level bonding with persons, organizations, and institutions beyond the face-to-face relationship. Within the primary group dimension studies commonly separate between horizontal cohesion (bonding with the small group) and vertical cohesion (bonding with direct commanders). Secondary group cohesion is likewise separated into two sub-dimensions: organizational and institutional cohesion. Organizational cohesion concerns bonding with the next higher organization unit (commonly the company), and institutional cohesion bonding with higher-level institutions such as the military and/or one’s military branch or specialty (Bury, 2018; Griffith, 2007; Salo, 2011; Siebold, 1999, 2007). Although related the four types of cohesion capture different relationships: all of which are nested within each other in a way similar to self-categorization theory’s view of super- and sub-ordinate identities within an organization (Hogg & Terry, 2000). A soldier is thus nested within each of these dimensions and has differing levels of cohesion in relation to each.

The SMMGC should be viewed as a conceptual whole (Siebold, 2011), with predictable relationships between dimensions, as well as sets of pre-theorized antecedents and outcome variables. A wide range of studies have found that the differing forms of cohesion are key variables in predicting performance, morale, motivation, and retention: to mention but a few outcome variables (Ahronson & Cameron, 2007; Bartone & Adler, 1999; Bartone et al., 2002; Griffith, 2007; Salo, 2011; Salo & Siebold, 2005, 2008). Theoretically the SMMGC can be seen as a well-established nomological network whose outcomes are important for military organizations. Despite its strong standing and bold theoretical claims there are, however, avenues for improvement.

First, although many empirical tests have evaluated the validity and reliability of the SMMGC few have evaluated full structural models of all dimensions. Studies mainly rely on calculating Cronbach’s alphas for internal validity and/or reduce measurements into composite variables using factor analysis for each separate dimension. Such methods tell us little of the empirical validity of the full model. Making use of empirical methods that can evaluate proposed dimensional structures is thus relevant to gain insight. It matters also for future research, as it has been claimed that “all four bonding levels (peer, leader, organizational, institutional) need to be measured, integrated, and included in research in the domain of cohesion” (Salo & Siebold, 2008). Those outside psychometrics agree that a fuller view is necessary (Käihkö, 2018). Second, although institutional bonding has long been theorized as the fourth dimension of unit cohesion (Bury, 2018; Salo, 2011; Salo & Siebold, 2008, 2005; Siebold, 2007), there is no agreed-upon conceptualization, and only few attempts at measurement (for example, see Salo, 2011; Salo & Siebold, 2005). Little has thus changed since Salo (2011) argued that secondary-group cohesion is overlooked. Additionally, seeing to the relatively little focused theoretical work, it is no surprise that items for measurement have been diverse and do not capture all of the concept’s sub-components. Below we synthesize previous research’s conceptualizations of institutional cohesion before suggesting measurements.

## Conceptualizing institutional cohesion

Institutional cohesion is commonly conceptualized as the second component of secondary group cohesion (the first being organizational cohesion), and as the process of social integration with larger military institutions to which soldiers belong, such as their military branch (Salo & Siebold, 2005). Organizational cohesion refers to similar processes but at a lower level of the military echelon, for instance the company level. At the level of organizational cohesion, soldiers know each other perhaps by name or face, but lack a deeper knowledge regarding individuals, and interactions are “intermittent and structured” and focused on predetermined roles and norms (Salo & Siebold, 2005). The organizational level provides specific missions and goals for its members and the bureaucratic and formal processes that provide material benefits (Siebold, 2007). Organizations exist, however, within a larger institution: the armed services or more specific yet still relatively abstract military branches or specialties.

In contrast to the organizational level, the institution, however, is linked to and embedded in larger society and culture, representing an intersection of military and civilian culture: the meso level of analysis. Salo and Siebold (2005) argue that “institutional bonding or cohesion occurs to the extent that there is a dependable exchange between the service members and the institution (i.e., service member effort, loyalty, and performance are traded for a sense of elevated purpose, compensation, systemic training opportunities, career progression, and social approved support for values, roles, careers, and way of life)” (p. 3). The institution is regarded as a component of the military system representing continuity and stability and which provides a “… general sense of purpose and meaningfulness …” (Siebold, 2007, p. 290). Salo (2011) argues (based on Cooley, 1909/1962) that institutional bonding is bonding with the distant secondary group (and organizational with the immediate secondary group), with which group members bond based on values, the mission, professional pride, and tradition. Institutional cohesion is thus more abstract than organizational cohesion’s mixture of abstract symbolism and concrete administrative/bureaucratic functions (Manning, 1991). Bonding with the institutional level is thus theorized to be based in affective rather than instrumental components, visible also in Gal’s (1985) view of institutional bonding as a form of conviction to the purpose of an institution.

Many have, additionally, argued that the institutional level supplies soldiers with a larger group identity to unite members from lower organizational levels (Ashforth, Johnson, Hogg, & Terry, 2001; Moskos, 1988; Salo, 2011). Siebold and Lindsay (1999) viewed institutional cohesion in much the same way, treating it mainly as an abstract positive identification with the army (“institutional integration”). Griffith, in early studies of unit cohesion (Griffith, 1988, 1989), identified a form of institutional cohesion he termed “sense of pride”. He theorized that his short battery captured the internalization of army values, a sense of pride in the army as an institution, and commitment to it. Viewed together, conceptualizations of institutional bonding have thus tried to capture a relatively broad range of related components which share a comparably abstract and affective-oriented nature.

While several theoretical components have been suggested, at least four are held in relative agreement. These are (1) identification with an institution, (2) pride in the institution, (3) positive evaluations of its functioning, and (4) commitment to the institution. Consequently, soldiers who strongly identify with the institution, take pride in it, positively evaluate its functioning, and are committed to it are those who demonstrate high institutional cohesion. Our concept of institutional cohesion is thus broader than organizational identification as identified by Mael and Tetrick (1992) but partly overlapping, since institutional cohesion also entails identification. We do acknowledge the ambiguity, since both cohesion and identification capture aspects of psychological attachment to the group but are operationalized differently across levels of the military hierarchy.^1^ However, we find merit in focusing on cohesion since we capture broader dynamics than identification and our concept is in line with previous studies trying to capture cohesion in a similar way. Actual empirical evidence of the content and function of this dimension is, however, scarce, as is possible empirical and conceptual overlap with organizational cohesion. In fact, only one study on a military sample (Salo, 2011) has actually attempted to measure this dimension within the SMMGC, with others implying measurement of it but using other overlapping or partial concepts (Griffith, 1989; Siebold & Lindsay, 1999). We, consequently, suggest a new measurement battery that we believe taps into the core of these four components.

## Methods

### Sample

We collected data from a sample of approximately 470 service members of the 2nd *Alpini* battalion: an elite unit specialty of the Italian army and its most deployed in kinetic operations. In Afghanistan they contributed to the multinational US-led counterinsurgency operation Enduring Freedom in 2002–03, followed by deployments in Regional Command Capital 2004–09, and Regional Command West near Herat. When surveyed the 2nd *Alpini* had returned 2 months earlier from a deployment with Task Force East, part of the International Security Assistance Force (ISAF). The deployment was not a war-fighting mission, but the context was highly volatile. Throughout its deployments in Afghanistan the contingent suffered six fatalities and more than a dozen injured in combat. Temporal proximity to the last deployment and in-field experience entails that we are using a militarily relevant sample.

Data were collected as part of a separate project where the goal was to understand the effect of mission perceptions on cohesion. An Italian-language pen and pencil questionnaire was distributed over 2 days on four different occasions. Participation was voluntary, but time had been allotted in daily schedules by commanders for us to inform respondents and hand out questionnaires. We offered no inducements for participation, beyond that the results of our study would be reported to the Italian army for possible improvements in the organization. All respondents were informed orally and in writing of their rights and complete anonymity offered. 457 out of 471 respondents gave their informed consents and completed the questionnaires (97% response rate). Our sample was 93% male, and 43% were between 22 and 29 years of age and 41% between 30 and 35. 33.5% were temporary recruits, 53% had permanent contracts, and 13% were commanding officers or NCOs. 63% were members of combat groups and the rest held administrative or logistical positions.

## Materials and methods

We now propose measurements for the four dimensions. We begin with institutional cohesion, before delineating the remaining three: all questions posed are available in the Appendix. In measuring the SMMGC it is important to operationalize the dimensions on different levels of the hierarchical structure of primary and secondary group cohesion. Horizontal cohesion was measured at the platoon level and vertical cohesion between platoon members and their platoon leader or next superordinate officer when the platoon structure was not applicable. Organizational cohesion was measured at the company-level. These are standardized approaches. Concerning institutional cohesion the theoretical literature is not as clear regarding what level of the hierarchy is “best” to operationalize on. Both the branch, the military at large, and “… bonding with higher hierarchical levels including up through the service itself” (Siebold, 2011) have been suggested. We chose to operationalize at the level of the Alpini as a specialty branch since this level in the hierarchy is separate enough from the company level (with regiments and brigades being found in between the two levels) so as to not confound the measurement from the outset, while simultaneously being a bearer of the values, traditions, and career paths deemed to be theoretically important for this type of cohesion.

It should also be noted that in terms of item selection we chose items aligned with the affective strand of cohesion (“social” as opposed to “task”; see Griffith and Vaitkus (1999)). This in order to align the four dimensions under study as institutional cohesion is geared toward the affective rather than the instrumental. To conduct a fair evaluation of institutional cohesion and its place among the other three dimensions it is necessary for all dimensions to be linked across the same theoretical axis. Focusing instead on, for instance, the task aspect of horizontal cohesion would make for a different type of comparison with institutional cohesion. This section also specifies covariates used to evaluate validity.

### Institutional cohesion

To measure institutional cohesion we constructed a six-item battery for use in the specific context. We draw heavily on measurements and theory constructed by Salo and Siebold (2005), Salo (2011), Siebold and Lindsay (1999), Mael and Tetrick (1992), and Griffith (1988, 1989) to capture the four theoretical components identified. We did not make use of all available items from these studies. For one, inclusion of all items would make for a long and inefficient battery. Second, many of the questions (particularly in Salo (2011) and Salo and Siebold (2005)) were not suitable for volunteer forces or mixed systems such as the Italian one, as they inquire regarding compulsory military service. We thus selected items deemed to tap into our proposed theoretical components and either used these directly or adapted them to context. An in-depth description of available items, inclusion and exclusion, and adaptations is available in the Appendix. The following questions were used:

**Table:** 

1. I am proud of being a member of the Alpini	4. I am an Alpino and I will remain one until I die
2. The Alpini is a very fine military institution in terms of operational performance	5. If someone criticizes the Alpini, it feels like a personal insult
3. All members of the Alpini share a special bond	6. The Alpini’s successes are my own successes

Question 1 was thought to tap directly into a sense of pride, questions 5 and 6 to tap identification, question 2 positive evaluations of functioning, and question 3 and 4 commitment to the institution: capturing all of our identified theoretical components. All items were scored on a 1–5 scale (from Strongly Disagree to Strongly Agree), where a 5 denotes higher levels of cohesion. Coefficient alpha was high at .92.

### Horizontal, vertical, and organizational cohesion

While institutional cohesion has not often been measured, this is not the case for the other three dimensions. To measure horizontal, vertical, and organizational cohesion we used only items with established validity from previous research. All scores were recorded on a scale of 1–5, ranging from Strongly disagree to Strongly agree. All the items used are available in the Appendix.

To measure horizontal cohesion we employed five items based on work by Salo (2011) and Jones et al. (2012). Cronbach’s alphas for these five items stood at .84. Example items are “I feel a sense of comradeship (or closeness) between myself and other people in my platoon” and “I feel appreciated in my platoon”. To measure vertical cohesion we used six items, stemming from work by Jones et al. (2012) and King, King, Vogt, Knight, and Samper (2006). Cronbach’s alpha was high at .81. Example items include “My platoon commanders do the following: Treat all members of the platoon fairly” and (reverse-coded) “Embarrass juniors in front of other platoon members”. To measure organizational cohesion five items from work by Salo (2011) were used: all geared toward cohesion with the company level. Cronbach’s alphas stood at .81. Example items are: “The atmosphere in my company is good” and “I would feel safe switching platoons in my company”.

### Covariates

Lastly, the questionnaire contained questions used to evaluate our cohesion variables’ validity. First, we inquired about perceived performance among the soldiers, asking them: “How would you rate your (squad/platoon) performance on your last mission to Afghanistan?” Performance was thus measured on two different levels of aggregation, scored on a scale of 1–4, from “Very bad” to “Very good”.^2^ Second, we inquired about the number of months each soldier had spent in his/her platoon. Third, we included a variable measuring the rank of each soldier. We scored the rank variable dichotomously where 0 equals soldiers and NCOs, and 1 denotes officers. Finally, we inquired into the soldiers’ levels of combat exposure, using an additive index derived from four indicators from Keane’s Combat Exposure Scale (Keane et al., 1989; scoring available in the Appendix). Expected relationships between cohesion dimensions and these variables are detailed in the Results section. Descriptive statistics for all variables are presented in Table 1.

**Table 1.** Descriptive statistics

Variable	Obs.	Mean (SD)	Min.–Max.
Rank	435	.07 (.26)	0–1
Time in platoon	414	27.4 (35.4)	1–192
Squad performance	329	3.5 (.57)	1–4
Platoon performance	325	3.4 (.65)	1–4
Combat exposure	457	1.3 (1.2)	1–5

### Statistical approach

To measure the validity and dimensionality of our concepts we use Confirmatory Factor Analysis (CFA). Compared to more traditional methods for evaluating dimensionality, internal reliability, and validity – such as coefficient alpha, principal components, and the creation of index variables for analysis – the CFA comes with several perks (Acock, 2013). For instance, coefficient alpha is highly sensitive to inflation due to the number of items, and is also not suitable for testing for dimensionality. Reliance purely on alpha scores can thus lead to faulty assumptions on measurement quality. Principal components analysis – while being able to account for dimensionality and data reduction – is unable to account for the covariance that items share within an underlying concept, thus (often erroneously) assuming that items lack unique variance. With a CFA one can isolate shared variance and thus discover what a series of questions share in measuring. Finally, CFAs (or, Structural Equation Models; SEM) provide more exact variables for prediction than index variables, as a CFA extracts and correlates only those portions of variables that are shared and thus measure underlying concepts. It thus parses out the measurement error inherent in the index variable-assumption that each item matters just as much as the next (*tau* equivalence, see Acock, 2013; Schmitt, 1996). To our knowledge the full four-factor SMMGC model has never been evaluated via a CFA framework. For interpretations of model fit we consistently rely on Hu and Bentler (1999) and Kenny (2014).

## Results

### Institutional cohesion as a stand-alone construct

We begin our analysis by evaluating institutional cohesion as a stand-alone construct. Recall that Cronbach’s alpha was high at .92, indicating high internal reliability. As argued above alpha scores are not, however, enough to prove measurement homogeneity or give the full story on data-to-theory fit, which we thus validate further via CFA (see Sundberg, 2016). We specified a CFA in which all six items were allowed to load on the presumed common latent variable (institutional cohesion) using maximum likelihood estimation. We additionally estimated the same type of CFAs for the remaining three dimensions of cohesion (horizontal, vertical, and organizational) for validation purposes. Fit statistics are shown in Table 2.

**Table 2.** Fit statistics for CFAs of the four separate dimensions

	(1) Horizontal cohesion	(2) Vertical cohesion	(3) Organizational cohesion	(4) Institutional cohesion
X^2^(*df*)	538.5(9)	28.9(9)	34.3(5)	92.9(9)
CFI	.96	.98	.97	.96
RMSEA	.12	.07	.08	.14
SRMR	.04	.03	.03	.03
Pclose	.000	.09	.001	.000
BIC	5655.8	6181.2	6460.2	7564.5
AIC	5717.1	253.8	6521.7	7638.7
N	439	415	447	456

The institutional cohesion model (Model 4) demonstrated only a decent fit to the data as a stand-alone construct. All items had strong loadings on the proposed latent variable of institutional cohesion (from .73 to 1.1), and key absolute measures of fit such as the Standardized Root Mean Square Residual (SRMR) and the Root Mean Square Error of Approximation (RMSEA) stood at .03 and .14, respectively, in all indicating a decent fit. The pclose (*p* of Close Fit, a hypothesis test that RMSEA is greater than .05) score was, however, significant. Taken together, the results are relatively promising as the test demonstrates strong loadings, decent fit to the data, unidimensionality in measurement, and strong internal reliability. The models specified for the remaining three dimensions (Models 1–3) were of decent to good fit. Viewed together, these four models demonstrate good internal reliability and acceptable initial validity.

### Distinguishing within secondary group cohesion

Next, we tested for discriminant and convergent validity (Adcock & Collier, 2001). In doing so we considered that institutional cohesion needs to be distinguishable from the closely related concept of organizational cohesion (discriminant validity). For institutional cohesion to be considered a separate dimension our items measuring organizational cohesion and institutional cohesion should prefer to be separated into two separate dimensions over loading on a single underlying dimension (secondary group cohesion): we want a two-dimensional model to fit the data better than a one-dimensional one. Additionally, we would theoretically expect some convergence in that the two concepts should be correlated (convergent validity). We tested these assumptions by first comparing the fits of two different CFAs, one in which all items for organizational and institutional cohesion are allowed to load on a single latent variable (which could be construed as being secondary group cohesion), and one in which we specify these items to load on separate but correlated latent variables (one institutional and one organizational dimension). We again used maximum likelihood estimation.

Our results demonstrate that all comparative fit indices show clear improvements when moving from a unidimensional (Model 5, *X*^2^ = 866.7, *df *= 44, RMSEA = .21, SRMR = .12) to a two-dimensional model (Model 6, *X*^2^ = 234.3, *df *= 43, RMSEA = .10, SRMR = .05). The Comparative Fit Index (CFI) improves from .74 to .94, and both AIC and BIC are significantly lower (suggesting a better fit) in the two-dimensional model (table available in online Appendix, Table A1). In a second test we nested the two models by – in one model – constraining the correlation between the two dimensions to be 1, and conducting a likelihood-ratio test. As chi-square differences were significant (*p* = .01) the two-factor model is the preferred choice also in this method. Consequently, our data prefers the two-dimensional model suggested by theory, rather than viewing the abstract secondary-group level as one-dimensional. As expected the two dimensions are highly correlated at .77, demonstrating their close relationship in the higher-order dimension. These models thus also speak to the validity of our construct, as well as the general organization of the higher-order dimension of secondary group cohesion.

### Full four-dimensional model

Lastly, we construct a number of tests and models to evaluate nomological validity: tests of expected correlations and relationships for a theoretical model’s structure (Adcock & Collier, 2001). In a first model we test the four-dimensional cohesion concept in full. As argued by for instance Siebold (2011) the SMMGC should be seen as modeling a conceptual whole, although divided into four dimensions and a separation between primary and secondary group cohesion. These theoretical dimensions and distinctions are thought to have empirical relevance and should thus be possible to capture when constructing an empirical model. It follows from the conceptualization of the SMMGC that our expectations – beyond a model that fits the data well – are that all four dimensions should correlate with one another but to differing degrees. Horizontal and vertical cohesion should be highly correlated as they are primary group dimensions, and organizational and institutional cohesion should continue to correlate highly as they belong to the secondary group. To test this expectation we specified a CFA in which all of our cohesion items were allowed to correlate with their respective theorized latent variables (horizontal, vertical, organizational, and institutional cohesion). These latent variables were then further specified to correlate with each other. We used, again, a maximum likelihood estimation.

Results demonstrated a model (*x*^2^, 502.6, df = 203) that fit the data well, with RMSEA and SRMR at .06 and .05, respectively, but with a significant pclose score (.005). All but two of the items loaded strongly on their respective latent variable (low loadings for one organizational cohesion item and one vertical). All four sub-dimensions were, as expected, correlated with each other (p.001), from a low of .28 (vertical cohesion to institutional cohesion), to a high of .79 (organizational cohesion to institutional cohesion). Institutional cohesion correlated with horizontal cohesion at .42. As expected, institutional cohesion is thus most strongly related to its secondary-group cousin in the form of organizational cohesion, and less related to the components of primary group cohesion. Results thus demonstrate that the proposed four-dimensional model fits the data well, and that the correlations between the four dimensions are as should be expected from the theoretical model.

### Prediction model

For institutional cohesion to be a useful concept – and our measurements of it valid – the construct should also be able to predict and be predicted by a range of variables posited to correlate with it (Adcock & Collier, 2001). Predictive power on outcome variables should also preferably be unique when controlling for other cohesion variables. As mentioned above the SMMGC provides a good basis for a nomological network even beyond expected correlations between dimensions. First, one of the most important outcomes posited by cohesion theorists to be positively predicted is performance: at least regarding the other three dimensions (for instance, Beal, Cohen, Burke, & McLendon, 2003; Siebold, 2007). Following from this relationship we could expect institutional cohesion to be positively and uniquely related to perceived performance. Second, cohesion tends to correlate with time spent with one’s comrades (Griffith & Vaitkus, 1999; Salo & Siebold, 2005; Siebold, 2007), although the causal direction is not easy to establish: either time spent builds cohesion, cohesion builds higher retention, or both. Irrespective we would expect institutional cohesion to predict the time (in months) a soldier has spent in his/her platoon. Third, we would expect institutional cohesion to correlate with the rank of each soldier, with soldiers higher up in the hierarchy more prone to demonstrate higher levels of institutional cohesion and identification (Ruffa & Sundberg, 2018). Fourth, cohesion is also often found to be heightened the more intense experiences the group has been through (Salo, 2011; Vaitkus & Griffith, 1990). We would not necessarily expect that this effect travels beyond the primary group where the bulk of, for instance, combat stressors are felt. Nevertheless, we include this test from the network to test the expectation of differing effects of exposure for differing dimensions. We tested these assumptions by allowing our latent variable (institutional cohesion) to predict rank, time in platoon, self-perceived performance during a six-month tour of Afghanistan (at the two levels of squad and platoon), and levels of combat exposure, while controlling for the effects of the other cohesion dimensions. Each outcome variable is tested in a separate model. Table 3 displays our results and a full correlation table for the variables included is available in the Appendix (Table A2).

**Table 3.** Predictive and discriminant validity of institutional cohesion

	(7) Rank	(8) Time in platoon	(9) Squadron performance	(10) Platoon performance	(11) Combat exposure
Institutional cohesion	.04 (.02)***	4.9 (2.2)**	.08 (.04)**	.18 (.05)***	.08 (.07)
Organizational cohesion	.001 (.02)	−.1 (2.5)	.005 (.04)	−.03 (.05)	−.4 (.09)***
Vertical cohesion	.03 (.04)	−2 (7.4)	.03 (.1)	.12 (.1)	−.33 (.09)
Horizontal cohesion	−.04 (.04)	6.6 (6.1)	.14 (.08)	.003 (.12)	.75 (.20)***
X^2^(*df*)	532.9 (221)	572.2 (221)	429.1 (221)	437.1 (221)	533.7 (221)
RMSEA	.06	.06	.06	.06	.06
SRMR	.05	.06	.05	.05	.05
pclose	.004	.000	.11	.07	.01
CFI	.93	.93	.94	.94	.94
n	385	372	303	301	404

N is smaller in these analyses than in the preceding ones, as not all members of the sample had been deployed. ***p > .01, **p > .05, *p > .10. Coefficients are unstandardized and standard errors are within parentheses.

In all five models in Table 3 our institutional cohesion construct demonstrated the expected correlations, even when controlling for the other cohesion dimensions. Higher levels of institutional cohesion predict higher scores of self-perceived performance for both performance measurements (M9 and M10). Institutional cohesion also positively predicted time in platoon (M8), with each one-step increase in institutional cohesion predicting an additional 4.9 months in a platoon (we make no judgment on the causal direction). Levels of institutional cohesion correlate positively with rank (M7), with higher levels of institutional cohesion being predictive of a soldier being an officer. Lastly, as expected, levels of combat exposure had no significant relationship with levels of institutional cohesion (M11). Horizontal cohesion, however, had a strong and significant relationship with this variable, which may be indicative of combat fusing stronger bonds at the primary group level. Taken together, our construct of institutional cohesion demonstrates relatively strong predictive validity within those parts of the nomological network that were testable with the data at hand.

In sum, as the data fit the proposed four-dimensional structure as well as it does, the empirics consequently demonstrate support for the proposed four-dimensional construct of cohesion. The close fit of the model shows how practically all indicators are strongly related to their proposed dimensions, and that these dimensions are empirically separate but correlated with each other in expected ways. Consequently, our analysis provides support not only for the existence of the dimension of institutional cohesion – and sound measurement of it – but also for the theorized overarching structure of the SMMGC.

## Discussion and conclusions

After empirical testing we demonstrate two main findings: (1) support for our conceptualization and measurement of institutional cohesion, and (2) further support for the validity and usefulness of the SMMGC in the form of a four-dimensional model of cohesion. That our proposed measurement of institutional cohesion was found to be valid and reliable is especially encouraging, as we proposed a measurement that could tap the four most important proposed components of the concept. Our study suggests that the SMMGC is a sound concept that manages to capture empirically both the micro and the meso-level dimensions of cohesion.

The above notwithstanding the SMMGC still lacks in at least three important ways on which future research should focus. First, further research should more clearly conceptualize and measure the macro level: perhaps along the lines drawn up by Salo (2011) and his thoughts on societal or national bonding. More systematic research on this topic is necessary seeing to how existing research highlights its possible importance (see also Käihkö, 2018). Research might here focus on how well an institution’s values and missions mesh with broader societal notions of the military and what this entails for levels of cohesion on the other levels. Second, there is also a need for a stronger conceptualization of the meso level. We believe especially that more work is necessary to grasp how/if the meso level can be separated from similar concepts such as organizational identification (Hogg & Terry, 2000; Mael & Tetrick, 1992), or if identification should be viewed squarely as one full component of this concept. Put differently, it can be argued that identification with an institution, pride in it, and perhaps also trust in its operational performance are all aspects of organizational identification. Should this be the case future work is necessary to resolve how institutional cohesion could be more precisely conceptualized and measured to avoid conceptual conflation. We would propose that future research should emphasize conceptually and try to measure empirically more instrumental bonds with the institutional level. One suggestion is to focus on institutions and career paths as well as rewards for performance. Focusing on instrumental bonds would also enable an operationalization of this dimension that more clearly overlaps with the other three in the model as these are often divided into affective and instrumental spheres. Third, for the SMMGC’s validity to be truly tested future research should attempt to apply it to non-Western and perhaps even non-state military forces: cross-cultural validity is crucial to evaluate the model’s universal claims of applicability (Käihkö, 2018; Käihkö & Haldén, 2019).

## Notes

1. We gratefully acknowledge one of the Reviewers for pushing us to clarify this point.

2. As noted by reviewers, self-ratings of performance can be construed as problematic indicators of actual battle-field or other task-related performance. While we agree with this critique, self-assessment is common in the literature (see, for instance, Oliver, Harman, Hoover, Hayes, and Pandhi (1998)). More important is the fact that we do not use these self-ratings to make any causal claims: only for reasons of testing validity. We admit that not making use of other types of data is a limitation that should be kept in mind.

## Appendix A

1. **Full list of items measuring cohesion**

***Horizontal cohesion***

How much do you agree or disagree with the following statements regarding your platoon?

**Table:**

1. I feel a sense of comradeship (or closeness) between myself and the other people in my platoon
2. I am able to go to most people in my platoon when I have a personal problem
3. I feel well informed about what is going on in my platoon
4. In combat, my platoon would help me even if it would put them in danger
5. I feel appreciated in my platoon

***Vertical cohesion***

My platoon commanders do the following:

**Table:**

1. Treat all members of the platoon fairly
2. Show concern about the safety of platoon members
3. Appreciate my service to the platoon
4. Were interested in what I thought and how I felt about things
5. Embarrass juniors in front of other platoon members
6. Accept extra duties or tasks for the platoon in order to impress their superiors

***Organizational cohesion***

**How much do you agree or disagree with the following statements regarding your company?**

**Table:**

1. I am proud of my company
2. The atmosphere in my company is good
3. I would feel safe switching platoons within my company
4. The commanding officers of my company are good and competent people
5. The company provides me with most things I need, both at work and off-duty

***Institutional cohesion***

**How much do you agree or disagree with the following statements regarding the Alpini?**

**Table:**

1. I am proud of being a member of the Alpini
2. The Alpini is a very fine military institution in terms of operational performance
3. All members of the Alpini share a special bond
4. I am an Alpino and I will remain one until I die
5. If someone criticizes the Alpini it feels like a personal insult
6. The Alpini’s successes are my own successes

2. **Discussion on item inclusion, exclusion, and modification**

As has been argued in the main manuscript attempts at measuring institutional cohesion have been few, as well as focused on measuring only specific sub-components of the theoretical concept. In creating the measurement applied in this study we first studied the theoretical literature to establish which sub-components had been theorized as part and parcel of the concept. Based on this list of at least four sub-components (identification, positive evaluations of performance, pride, and commitment) we subsequently perused the literature to select indicators for these components, always keeping efficiency in mind so as to not create a too long battery. Items are drawn from or inspired by items from Salo and Siebold (2005), Salo (2011), Siebold and Lindsay (1999), Mael and Tetrick (1992), and Griffith (1988; 1989).

The most ambitious attempts to measure institutional cohesion come from Salo (2011) and Salo and Siebold (2005). They applied the following questions:

1. I am not interested in military service.

2. I feel at home in military service.

3. All men should carry out military service as a part of total defence.

4. Military service is every male citizen’s duty.

5. I have considered applying to civilian service.

6. I have considered dropping out of service.

We made no direct use of these questions for our measurement, but drew inspiration from them and their theoretical discussions on institutional cohesion. The main reason for excluding these questions is based on their construction for use in the Finnish conscript system where military service is compulsory for all males. Many of the questions are thus related explicitly to this military system and what one feels about it (e.g. total defence, applying instead to civilian service etc). Such system-dependent questions were deemed to not be able to travel well to other contexts. Additionally, we questioned whether the level of analysis of these questions was suitable for measuring institutional cohesion as the referent object is most often military service as a concept rather than the army or the military institution itself. We drew inspiration from Salo’s and Siebold’s questions and theoretical discussions, however, to create Q3 (‘All members of the Alpini share a special bond’) to capture bonding with and commitment to the institution and its members (mainly feeling at home and doing one’s duty in their battery).

Siebold and Lindsay (1999) created an army identification scale, parts of which were included in our battery to measure the identification component of institutional cohesion. Their battery was as follows:

Army Identification Scale

20. When someone criticizes the Army, it feels like a personal insult.

21. I’m interested in what others say about the Army.

22. When I talk about the Army, 1 usually say ***we*** instead of ***they.***

23. The Army’s successes are my successes.

24. When someone praises the Army, it feels like a personal compliment.

From this battery we opted to use Q20 and Q23 as these appeared to be the most strongly linked to a process of identification. These two items are also part of Mael and Tetrick’s (1992) battery of questions for organizational identity and thus seemed to be sound choices for this sub-component. We adapted these two questions to create Qs 5 and 6, changing only the referent object to Alpini.

In work by Griffith he has on several occasions wanted to measure sense of pride and commitment. From his 1988 work we adapt his Sense of Pride question from “I am proud of my company” to create our Q1, querying “I am proud to be a member of the Alpini”. In the same 1988 piece Griffith also uses the question “What I do in the Army is worthwhile”. Due to efficiency we chose to use only the first question, as this asks directly about pride.

In his 1999 work Griffith again measures Sense of Pride, but also Commitment to the Army. He poses a question asking “Do you want to serve in this unit after your initial enlistment?”. This question appears a solid measurement of commitment to an organization. We adapated it, however, to (1) fit the mixed Italian military system (some conscription and some career soldiers), and (2) to capture a deeper commitment than one single enlistment or tour of duty. Our question (Q4) subsequently reads “I am an Alpino and will remain one until I die”.

Finally, we could not identify any sound measurements of positive evaluations of the function of the institution. We thus created Q2 (“The Alpini is a very fine military institution in terms of operational performance”) ourselves to tap into this dimension.

**Table A.1.** Unidimensional and two-dimensional model of secondary-group cohesion

	(5) Unidimensional Model	(6) Two-dimensional Model
X^2^(*df*)	866.7(44)	234.3(43)
CFI	.74	.94
BIC	14306-4	13815.6
AIC	14441.7	13676.2
RMSEA	.21	.1
SRMR	.12	.05

3. **Measuring combat exposure**

To measure the concept of combat exposure we used four items from Keane’s Combat Exposure Scale deemed relevant (Keane, Fairbank, Caddell, Zimering, Taylor, and Mora 1989). These four items were: ‘When you were in Afghanistan, how often were you involved in the following? (1) firing upon the enemy? (2) were you fired upon? (3) did you sustain casualties or fatalities in combat or combat-like situations? (4) did you conduct patrols in dangerous or hostile territory? Response categories varied from 0 (Never) to 5 (16 times or more). These four items showed an acceptable construct validity of .76. Means across these four items were used in the analyses in the form of an additive index.

**4.**

**Table A2.** Correlation table

	1	2	3	4	5	6	7	8	9
1. Horizontal cohesion	-	-	-	-	-	-	-	-	-
2. Vertical cohesion	.**78**	-	-	-	-	-	-	-	-
3.Organizational cohesion	.**50**	.**55**	-	-	-	-	-	-	-
4. Institutional cohesion	.**50**	.**32**	.**59**	-	-	-	-	-	-
5. Rank	.07	.1	.**15**	.**23**	-	-	-	-	-
6. Time in platoon	.**16**	.**11**	.**10**	.**22**	-.01	-	-	-	-
7. Squadron performance	.**27**	.**23**	.**21**	.**25**	.**17**	-.04	-	-	-
8. Platoon performance	.**23**	.**21**	.**21**	.**33**	.**13**	-.09	.**75**	-	-
9. Combat exposure	.**15**	-.03	**-.17**	.04	.001	.08	.03	.10	-

*Note*: Bold denotes p>.05
